# Association between healthy vascular aging and the risk of the first stroke in a community-based Chinese cohort

**DOI:** 10.18632/aging.102170

**Published:** 2019-08-15

**Authors:** Yingying Yang, Anxin Wang, Xiaodong Yuan, Quanhui Zhao, Xiaoxue Liu, Shuohua Chen, Xiuyan Wang, Yongjun Wang, Shouling Wu, Yilong Wang

**Affiliations:** 1 Department of Neurology, Beijing Tiantan Hospital, Capital Medical University, Beijing, China; 2 Advanced Innovation Center for Human Brain Protection, Capital Medical University, Beijing, China; 3 Department of Neurology, Kailuan General Hospital, North China University of Science and Technology, Tangshan, China; 4 Graduate School, North China University of Science and Technology, Tangshan, China; 5 Department of Cardiology, Kailuan General Hospital, North China University of Science and Technology, Tangshan, China; 6 Department of Cardiology, Tangshan People’s Hospital, North China University of Science and Technology, Tangshan, China; 7 Department of Geriatric Disease, Kailuan General Hospital, North China University of Science and Technology, Tangshan, China

**Keywords:** healthy vascular aging, stroke, arterial stiffness, pulse wave velocity

## Abstract

In this study we tested whether vascular aging is associated with the risk of first stroke in the Kailuan cohort, a community-based Chinese cohort. For participants aged ≥ 50 years, healthy vascular aging (HVA) was defined as an absence of hypertension and a brachial-ankle pulse wave velocity < the mean + 2 standard deviations, which was determined from a reference sample of healthy participants aged < 30 years. The primary outcome was first stroke (ischemic or hemorrhagic). In total, 11,474 participants were enrolled. The prevalence of HVA decreased from 36.0% in participants aged 50-59 years to 4.7% in those aged ≥ 70 years. During a median follow-up of 3.3 years, the incidence of first stroke was 0.5% in the HVA group but was 2.6% in the Non-HVA group. After adjusting for confounding variables, HVA was associated with a 0.32-fold lower risk of first stroke compared to the Non-HVA group (95% confidence interval, 0.18-0.56; *p* < 0.001). It thus appears that HVA reduced the risk of first stroke in a community-based Chinese population. This suggests that evaluation of vascular aging as part of public health screening may be useful for stroke risk assessment.

## INTRODUCTION

Age-related alterations in blood pressure related to vascular aging is attributable primarily to increases in the stiffness of the large elastic arteries and to endothelial dysfunction [1–3]. Although such vascular aging may seem inevitable, several studies have shown that it is uncommon in some populations leading healthy lifestyles, indicating that it can be avoided [4–6]. Moreover, various pharmacological interventions and lifestyle modifications that can influence blood pressure and arterial stiffness are promising de-stiffening therapies that may prevent or delay vascular aging [7–11].

Healthy vascular aging (HVA), a new concept defined as the absence of age-related hypertension and increased arterial stiffness, has been shown to be associated with a reduced risk of cardiovascular disease in Western populations [12, 13]. We therefore speculated that HVA may be related to a reduced risk of first stroke. To test that idea, we investigated the association between HVA and the risk of first stroke in community-based Chinese cohort.

## RESULTS

### Study participants

**Figure 1:**
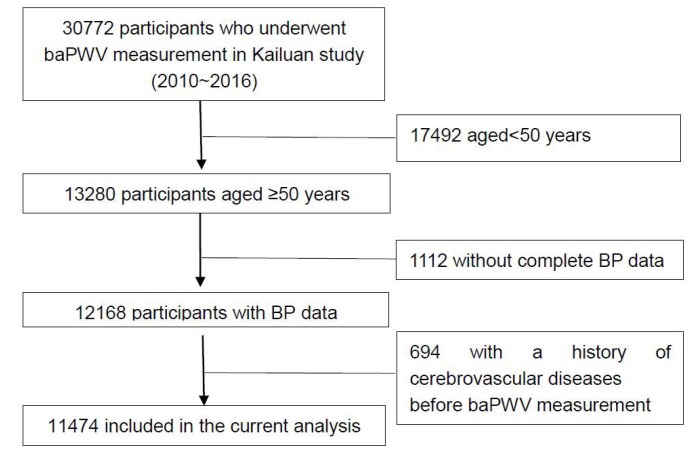
**Flow diagram of the participant selection in the current analysis.** Abbreviations: BP, blood pressure; baPWV, brachial-ankle pulse wave velocity.

From January 1, 2010 to December 31, 2016, brachial-ankle pulse wave velocity (baPWV) measurements were made in 30,772 participants in the Kailuan cohort. Among them, 13,280 were older than 50 years. After excluding participants for whom blood pressure data was lacking (N=1112) and those with a history of cerebrovascular diseases before the baPWV measurement (N=694), a total of 11,474 participants were included in the study (Figure 1). Among that group, 3003 (26.2%) exhibited HVA at baseline, whereas the remaining 8471 (73.8%) did not. A comparison of demographic and clinical information between the HVA and Non-HVA groups is shown in Table 1.

**Table 1:** Baseline characteristics of participants included in the study.

**Variable**	**Non-HVA group (n=8 471)**	**HVA group (n=3 003)**	***p***
**Age(y)**	61.8±8.9	56.3±5.7	<0.001
50y≤age<60y	4136(48.8)	2324(77.4)	
60y≤age<70y	2679(31.6)	597(19.9)	
age≥70y	1656(19.5)	82(2.7)	
Female	1981(23.4)	895(29.8)	<0.001
SBP(mmHg)	140.7±18.3	118.8±10.6	<0.001
DBP(mmHg)	87.0±11.0	77.2±6.7	<0.001
Heart rate(bpm)	75.0±11.5	71.4±10.1	<0.001
baPWV(m/s)	18.0±3.5	13.4±1.1	<0.001
**Cardiovascular risk factors**			
BMI(kg/m^2^)	25.5±3.3	24.5±3.0	<0.001
Current smoking	2640(31.2)	1005(33.5)	0.020
Current alcohol drinking	880(10.4)	260(8.7)	0.020
Inactive physical activity	1668(19.7)	500(16.7)	0.001
History of hypertension	6035(71.2)	0(0)	<0.001
History of diabetes	2349(27.7)	320(10.7)	<0.001
History of hyperlipidemia	4797(56.6)	1482(49.4)	<0.001
Monthly income ≥ $120	490(5.8)	154(5.1)	0.310
Education (≥High school)	1703(20.1)	789(26.3)	<0.001
**Laboratory results**			
FBG(mmol/L)	6.5±2.7	5.7±1.6	<0.001
TC(mmol/)	5.3±1.8	5.2±1.4	<0.001
LDL-C(mmol/L)	2.7±1.0	2.6±1.0	0.002
HDL-C(mmol/L)	1.5±0.8	1.5±0.5	<0.001
Hs-CRP(mg/L)	1.4(0.7-2.9)	1.10(0.6-2.2)	<0.001
**Medication use**			
Antihypertensive drugs	2464(29.1)	0(0)	<0.001
Antidiabetic drugs	903(10.7)	109(3.6)	<0.001
Lipid-lowering drugs	129(1.5)	20(0.7)	0.010

Abbreviations: HVA: healthy vascular aging; SBP: systolic blood pressure; DBP: diastolic blood pressure; baPWV: brachial-ankle pulse wave velocity; BMI: body mass index; FBG: fasting blood glucose; TC: total cholesterol; LDL-C: low-density lipoprotein cholesterol; HDL-C: high-density lipoprotein cholesterol; hs-CRP: high-sensitive C-reactive protein.

### The prevalence and determinants of HVA

**Figure 2:**
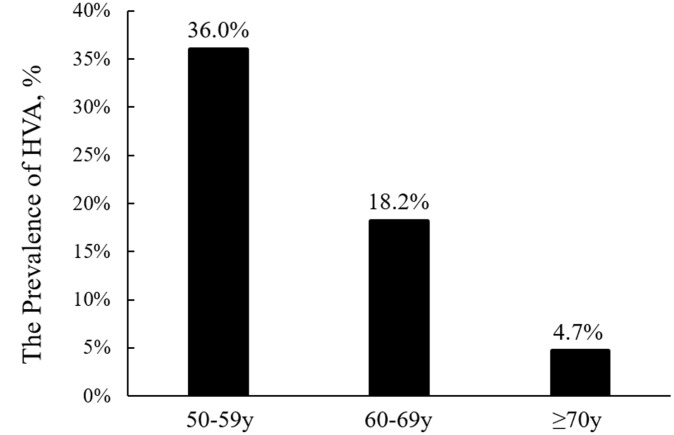
**Comparison of the prevalence of healthy vascular aging (HVA) among different age groups.**

The prevalence of HVA was 36.0% among participants aged 50 to 59 years, 18.2% among participants aged 60 to 69 years, and 4.7% among those aged ≥70 years (Figure 2). Using a multivariable analysis, we found that younger age, female sex, lower body mass index (BMI), lower high-sensitive C-reactive protein (hs-CRP), lower fasting blood glucose (FBG), higher education level, active physical activity, absence of current alcohol drinking, and the absence of diabetes mellitus and hyperlipidemia were significantly associated with HVA (*p*<0.05) (Table 2).

**Table 2:** Multivariate logistic regression HVA vs. Non-HVA group.

**Variable**	**OR for HVA (95%CI)**	**p**
Age	0.90(0.89–0.91)	<0.001
Male	0.685(0.61–0.77)	<0.001
BMI	0.91(0.90–0.93)	<0.001
Hs-CRP	0.99(0.97–1.00)	0.013
FBG	0.92(0.88–0.96)	<0.001
Inactive physical activity	0.87(0.77–0.99)	0.031
Education (≥High school)	1.22(1.10–1.36)	<0.001
Current alcohol consumption	0.78(0.66–0.91)	0.002
History of diabetes	0.61(0.50–0.74)	<0.001
History of hyperlipidemia	0.84(0.75–0.93)	0.007

Abbreviations: HVA: healthy vascular aging; BMI: body mass index; Hs-CRP: high-sensitive C-reactive protein; FBG: fasting blood glucose; OR: odds ratio; CI: confidence interval.

### Association between HVA and the risk of first-ever stroke

**Figure 3:**
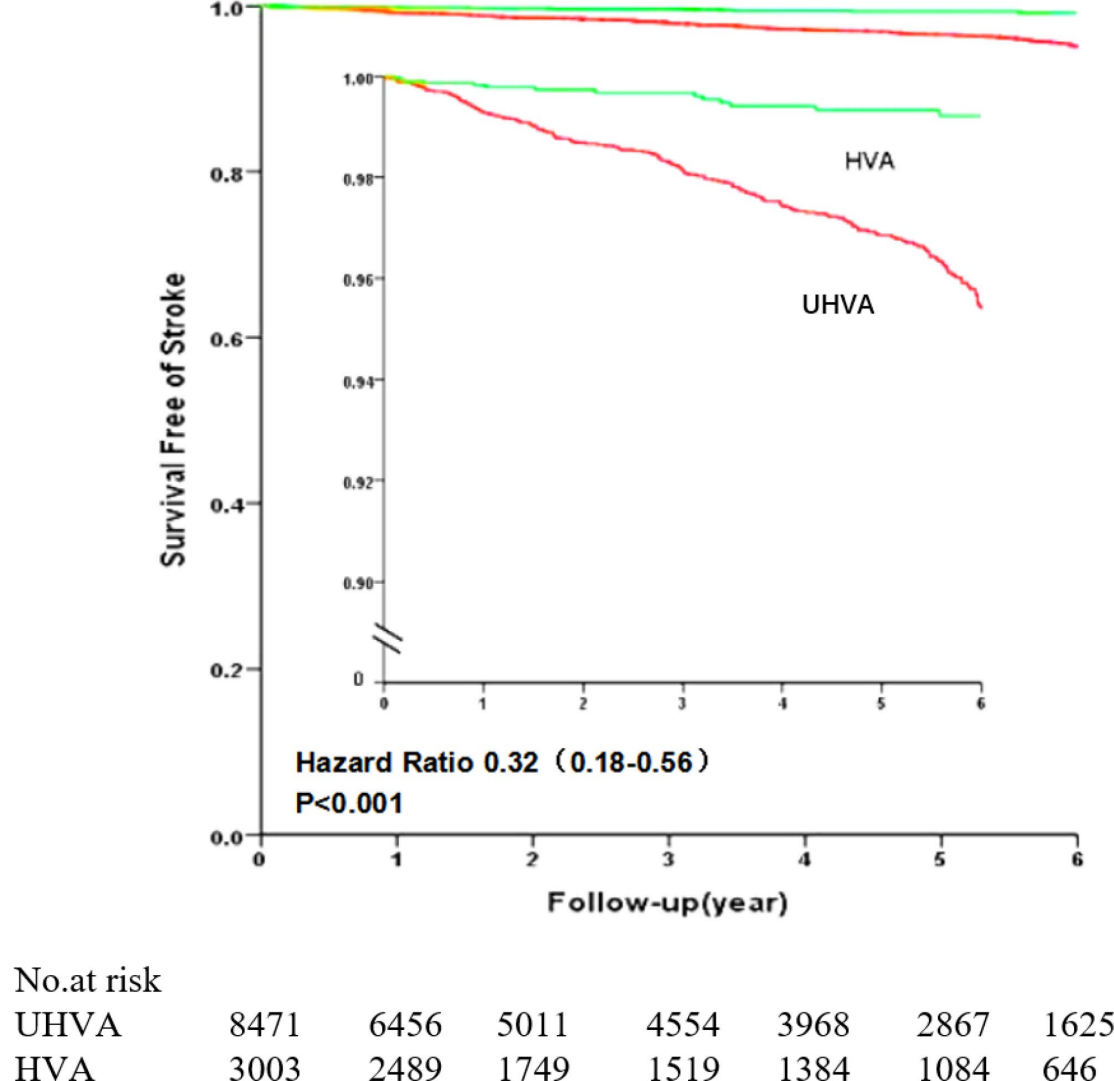
**Comparison of stroke-free survival between the HVA and Non-HVA groups.** The inset shows the same data on an enlarged segment of the y-axis. Abbreviations: HVA: healthy vascular aging.

The follow-up times were similar in the two groups (HVA group, 3.37 ± 2.25 years; Non-HVA group, 3.29 ± 2.31 years). During a median follow-up of 3.3 years, first stroke occurred in 0.5% (15/3003) of participants in HVA group, as compared to 2.6% (217/8471) of those in the Non-HVA group (*p*<0.001). First ischemic stroke occurred in 0.5% (14/3003) of participants in the HVA group, as compared to 2.3% (198/8471) of those in the Non-HVA group (*p*<0.001) (Table 3). Participants with HVA had considerably lower age and sex-adjusted (model 1) risk of stroke (hazard ratio (HR), 0.21; 95% confidence intervals (CI), 0.12-0.36) and ischemic stroke (HR, 0.22; 95% CI, 0.12-0.37) as compared to participants in the Non-HVA group (Table 4). After adjusting for other cardiovascular risk factors (including BMI, smoking, alcohol consumption, physical activity, history of diabetes, history of hyperlipidemia) (model 2), HVA was associated with a 0.23-fold (95% CI, 0.13–0.39) lower risk of stroke and a 0.23-fold lower risk (95% CI, 0.14–0.41) of ischemic stroke. After further adjusting for monthly income, education level, FBG, total cholesterol (TC), low-density lipoprotein cholesterol (LDL), hs-CRP, and use of anti-diabetic and lipid-lowering medications (model 3), HVA was associated with a 0.23-fold (95% CI, 0.14-0.40) lower risk of stroke and a 0.24-fold lower risk (95% CI, 0.14–0.42) of ischemic stroke. After additionally adjusting for systolic blood pressure (model 4), HVA was associated with a 0.32-fold (95% CI, 0.18-0.56) lower risk of stroke and a 0.32-fold lower risk (95% CI, 0.18-0.58) of ischemic stroke (Figure 3).

**Table 3:** Outcome incidence during follow-up.

**Outcomes**	**Non-HVA (n=8471)**	**HVA (n=3003)**	***p* value**
Total stroke (%)	217(2.6)	15(0.5)	<0.001
Ischemic stroke (%)	198(2.3)	14(0.5)	<0.001
Hemorrhagic stroke (%)	28(0.3)	1(0)	0.003

Abbreviation: HVA: healthy vascular aging.

**Table 4:** Risk of first stroke or ischemic stroke related to HVA vs Non-HVA.

**Outcome**	**HR(95%CI)**	***p* value**
Stroke		
Model 1	0.21(0.12–0.36)	< 0.001
Model 2	0.23(0.13–0.39)	< 0.001
Model 3	0.23(0.14–0.40)	< 0.001
Model 4	0.32(0.18–0.56)	< 0.001
Ischemic stroke		
Model 1	0.22(0.12–0.37)	<0.001
Model 2	0.23(0.14–0.41)	<0.001
Model 3	0.24(0.14–0.42)	<0.001
Model 4	0.32(0.18–0.58)	<0.001

Model 1: Adjusted for age and sex.

Model 2: Model 1 + adjustment for body mass index, smoking, alcohol drinking, physical activity, history of diabetes, history of hyperlipidemia.

Model 3: Model 2 + adjustment for monthly income, education level, fast blood glucose, total cholesterol, low-density lipoprotein cholesterol, high-sensitive C-reactive protein, use of antidiabetic and lipid-lowering medications.

Model 4: Model 3 + adjustment for systolic blood pressure

Abbreviations: HVA: healthy vascular aging; HR: hazard ratio; CI: confidence interval.

## DISCUSSION

The main finding of this study was that HVA was significantly associated with a lower risk of first stroke in a community-based Chinese cohort. Although the relationship between arterial stiffness and blood pressure is complex [14–18], the combination of normal pulse wave velocity and an absence of hypertension may be meaningful for prediction of cardiovascular disease risk. HVA was previously evaluated using two easily obtained metrics of vascular health: blood pressure and pulse wave velocity [12]. Considering the racial and geographic differences between Western and Chinese populations, our study builds a Chinese reference sample and establishes normal baPWV values for defining HVA, instead of adopting the reported references from a Western community [19]. Comparing the results from the Framingham heart study with our findings, we find that the prevalence of HVA was higher in this Chinese population than in the Western population (26.2% vs. 17.7%), particularly for participants aged 60 to 69 years (18.2% vs. 7.4%). Data from both the Framingham and Kailuan studies show that the prevalence of HVA decreases sharply with age. HVA appears achievable but is rare among participants aged ≥ 70 years. This may be attributable to greater arterial stiffness and a higher prevalence of hypertension among elderly participants. Our study also suggests that HVA status may be influenced by both non-modifiable (age and sex) and modifiable (BMI, hs-CRP, FBG, physical activity, alcohol consumption, education level, history of diabetes mellitus and hyperlipidemia) factors [20, 21]. Notably, in a univariate analysis, the fraction of participants currently smoking was higher in the HVA group than in the Non-HVA group. However, we did not observe an independent association between smoking status and HVA after multivariable logistic regression, which is consistent with an earlier study [12]. In addition, some epidemiologic studies have observed that smokers might have lower rates of hypertension and decreased arterial stiffness [22, 23]. Therefore, the impact of smoking on vascular health remains unclear and seems not straightforward.

The large sample size in our study increases its statistical reliability. In addition, all of the participants had access to local medical insurance, which guaranteed that the outcome data could be tracked through the medical insurance system. Nevertheless, several limitations should be noted. First, to evaluate the arterial stiffness, baPWV was adopted instead of carotid-femoral pulse wave velocity (cfPWV) [24–26]. However, because of the simplicity of its measurement and good correlation with cfPWV, we suggest baPWV could be potentially applied in clinical practice [27–29]. Second, participants in the Kailuan cohort volunteered to undergo baPWV measurement, which could potentially introduce selection bias. Third, since the Kailuan Group is a coal energy and chemical enterprise, males make up the majority of employees. All employees (≥ 18 years) were enrolled in the Kailuan cohort, which led to a gender imbalance among the participants in the analysis (males, 75%; females, 25%). Finally, because the numbers of incident strokes in this community-based population were limited, we did not conduct a stratification analysis based on age. In the future, the follow-up will go on to determine whether the effect of HVA is similar in different age groups.

In conclusion, it appears that HVA may have reduced the risk of first stroke in a community-based Chinese cohort. This suggests that evaluation of vascular aging as part of public health screening may be useful for stroke risk assessment and for stroke prevention.

## MATERIALS AND METHODS

The data supporting the findings of this study are available from the corresponding author upon reasonable request.

### Study participants

The Kailuan cohort is an ongoing prospective cohort based in the Kailuan community in Tangshan City in the north of China [30, 31]. All employees (≥ 18 years) of the Kailuan Group, a coal energy and chemical enterprise, were enrolled with the aim of investigating the risk factors of various diseases (e.g., stroke and cancers). As of December 31, 2016, 171,414 participants were included in the Kailuan cohort. The study’s protocol was approved by the participating hospitals’ ethics committees. All participants provided written informed consent. All participants underwent questionnaire assessments, clinical examinations and laboratory tests at the 11 hospitals responsible for healthcare in the Kailuan community.

To capture a distinct sample of individuals with HVA, in the present study we considered only participants in late middle age or older (aged ≥ 50 years), and we only enrolled participants who underwent baPWV measurement between 2010 and 2016. We excluded participants for whom blood pressure data was lacking as well as those with a history of cerebrovascular diseases before the baPWV measurement.

### Assessment of baPWV

BaPWV was measured using a BP-203 RPE III networked arteriosclerosis detection device produced by Omron Health Medical Co., LTD (China) [30, 31]. Participants received baPWV assessment after 5 min of rest in a supine position. Cuffs were applied to both arms and ankles. The lower edge of the arm cuff was positioned 2-3 cm above the cubital fossa transverse striation, and the lower edge of the ankle cuff was positioned 1-2 cm above the medial malleolus. The heartbeat monitor was placed at the left edge of the sternum, and electrocardiogram electrodes were placed on both wrists. BaPWV was calculated as the distance between the two sites divided by the pulse transit time (defined as the time interval between the wave fronts of the brachial and ankle waveforms). The distance between the sampling points was calculated automatically according to the subject’s height. The maximum value of the bilateral baPWV was used in our study. Using the detection device, we were able to read the baPWV value directly. The methodology for baPWV measurement was the same for all participants.

Baseline data on demographics and cardiovascular risk factors were collected through face-face interviews when baPWV was measured. The collected data included age, sex, BMI, smoking, alcohol consumption, physical activity, history of diabetes, history of hyperlipidemia, monthly income, education level, FBG, TC, LDL, hs-CRP, use of antidiabetic or lipid-lowering drugs. Physical activity was evaluated from responses to questions about the type and frequency of physical activity at work and during leisure time. Inactive physical activity was defined as no physical activity or less than 30 minutes every week.

### Assessment of HVA

**Figure 4:**
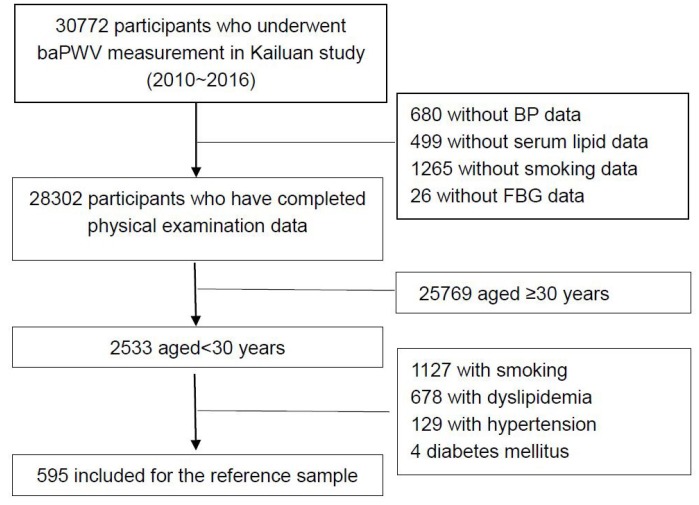
**Flow diagram of the participant selection for determination of the mean baPWV value.** Abbreviations: baPWV: brachial-ankle pulse wave velocity; BP: blood pressure; FBG: fasting blood glucose.

We defined HVA for participants aged ≥ 50 years as follows [12]: 1) absence of hypertension, which was defined as a systolic blood pressure < 140 mmHg, a diastolic blood pressure < 90 mmHg (blood pressure was measured at least twice at different occasions), and an absence of antihypertensive drugs; 2) baPWV < mean + 2 standard deviations (SD), which was determined from a reference sample of healthy participants aged 18-30 years with no cardiovascular risk factors (e.g., hypertension, diabetes, smoking, hyperlipidemia) and without a history of myocardial infarction, stroke, and other cardiovascular disease. To obtain the reference value for baPWV, we enrolled 595 healthy participants aged < 30 years from the Kailuan study (Figure 4). The results showed that the reference value for baPWV was 11.90 ± 1.65 m/s. Thus, HVA was defined as a normotensive blood pressure and baPWV < 15.20 m/s. Based on these data, participants were classified into HVA and Non-HVA groups.

### Outcome assessment and follow-up

The participants were followed up by face-to-face interviews at every two-year routine medical examination until December 31, 2017 or to death. The outcome information for the participants without face-to-face follow-up was obtained by referring to death certificates from provincial vital statistics offices, discharge summaries from the 11 hospitals, and medical records from medical insurance. The follow-ups were performed by hospital physicians, research physicians, and research nurses who were blinded to the baseline data. All stroke records were reviewed by two independent stroke specialists. If there were instances of disagreement in a single case, the final evaluation was made by the event adjudication committee.

The primary outcome was the first occurrence of stroke (both ischemic and hemorrhagic). These included the first nonfatal stroke event or stroke death without a preceding nonfatal event. Stroke was diagnosed according to the World Health Organization criteria [32] combined with brain computed tomography (CT) or magnetic resonance (MR) imaging for confirmation. The secondary outcome was the first occurrence of ischemic stroke. All study outcomes were validated by the Data Safety Monitoring Board and the Arbitration Committee for Clinical Outcomes.

### Statistical analysis

Continuous variables are presented as the mean ± SD or median with interquartile range, while categorical variables are presented as frequencies with percentages. The nonparametric Wilcoxon or t tests were used to compare group differences for continuous variables and χ^2^ tests were used for categorical variables.

The association between baseline characteristics and HVA was investigated using multivariate backward logistic regression, and variables with *p* values < 0.05 were selected as confounding variables. Odds ratios (ORs) with the 95% CI were reported. We also examined associations between HVA and first stroke or ischemic stroke using Cox proportional hazards regression models. For each outcome, 4 multivariable models were performed. In model 1, we only adjusted for age and sex. In model 2, we additionally adjusted for other cardiovascular risk factors (including BMI, smoking, alcohol consumption, physical activity, history of diabetes, history of hyperlipidemia). In model 3, we further adjusted for monthly income, education level, FBG, TC, LDL, hs-CRP, and use of antidiabetic or lipid-lowering medications. In model 4, we further adjusted for systolic blood pressure. Life table methods were used to calculate the cumulative rate of stroke-free survival, and a curve was generated.

Two-sided *p* values < 0.05 were considered significant. All analyses were performed using SAS 9.4 (SAS Institute, Cary, North Carolina).
